# Possibility of Human Epidermal Growth Factor Receptor 2 Expression as a Treatment Selection Indicator in Early Triple-Negative Breast Cancer

**DOI:** 10.7759/cureus.74561

**Published:** 2024-11-27

**Authors:** Namiko Tanaka, Seiichi Imanishi, Chisato Takeuchi, Takayoshi Goto, Nobuyoshi Kittaka

**Affiliations:** 1 Department of Breast Surgery, Osaka Rosai Hospital, Osaka, JPN; 2 Department of Diagnostic Pathology, Osaka International Medical &amp; Science Center, Osaka, JPN

**Keywords:** early triple-negative breast cancer, her2, immune checkpoint inhibitor, neoadjuvant chemotherapy, prognosis

## Abstract

Background

This study aimed to evaluate the relationship among human epidermal growth factor receptor 2 (HER2) expression level, pathological complete response (pCR) rate of neoadjuvant chemotherapy, and prognosis in early-stage triple-negative breast cancer (TNBC).

Methodology

This retrospective study analyzed the relationship among HER2 expression level, pCR rate, clinicopathological factors, and prognosis in 39 patients who were diagnosed with TNBC between 2012 and 2020 at Osaka Rosai Hospital and underwent surgery after neoadjuvant chemotherapy (NAC).

Results

Patients’ age ranged 33-86 (median = 57) years, and the observation period ranged 5-130 (median = 60) months. The HER2 expression levels were HER2 (0), HER2 (1+), and HER2 (2+, fluorescence in situ hybridization test (FISH); negative) for 18, 12, and 9 cases, respectively. The pCR rates were 38.9%, 8.3%, and 44.4% for HER2 (0), HER2 (1+), and HER2 (2+, FISH; negative), respectively, and no correlation was observed with the degree of HER2 expression. The prognosis of distant disease-free survival (DDFS) differed depending on the HER2 status (p = 0.032), and this trend was also observed in overall survival (OS) (p = 0.012). This tendency became even stronger when comparing HER2-low and HER2 (0) (p = 0.028 and p = 0.01, respectively). HER2 expression was significantly decreased from before to after NAC (p = 0.001).

Conclusions

HER2 expression did not correlate with the pCR rate of NAC but did correlate with DDFS and OS. Thus, patients with an HER2 (0) status are considered to have a poor prognosis and should be more aggressively considered for perioperative chemotherapy, e.g., immune checkpoint inhibitors.

## Introduction

Breast cancer is the most common cancer among women and ranks second in cancer-related deaths among women [1]. Triple-negative breast cancer (TNBC) accounts for 10%-15% of breast cancers and still has a poor prognosis compared with other subtypes [2-5]. The use of pembrolizumab, an immune checkpoint inhibitor, has been expanded in the perioperative period for early-stage TNBC and is expected to improve prognosis [6]. However, the prognosis is good even without the use of immune checkpoint inhibitors if the pathological complete response (pCR) is achieved by preoperative neoadjuvant chemotherapy (NAC) consisting of anthracycline and taxane (A/T), which is the conventional standard treatment [7-9]. However, no consensus has been established as to which patients with early-stage TNBC can be treated with NAC consisting of A/T to achieve pCR. Immune checkpoint inhibitors carry the risk of developing immune-related adverse events that are difficult to manage [6,10]. We do not have sufficient insights into determining which groups require immune checkpoint inhibitors for prognosis and which groups may be satisfied with conventional A/T chemotherapy. On the contrary, the expression levels in cases previously diagnosed as human epidermal growth factor receptor 2 (HER2)-negative were subdivided into HER2 (0), HER2 (1+), and HER2 (2+, FISH; negative). HER2 (1+) and HER2 (2+, fluorescence in situ hybridization (FISH); negative) are now defined as HER2-low [11]. The indications for trastuzumab deruxtecan have been expanded for HER2-low advanced and recurrent breast cancer, and an improved prognosis was reported [12]. Although several studies have reported the prognosis of TNBC depending on the HER2 status, no consensus has been reached [13-17]. In this study, we investigated how HER2 status and clinicopathological factors influence the prognosis of patients with TNBC treated with NAC consisting of A/T. On the contrary, studies have reported that the expression of interleukin-34 (IL-34) not only is a poor prognosis indicator in TNBC [18] but also may reduce the effectiveness of immune checkpoint inhibitors [19]. However, no studies have reported yet on the relationship between ERBB2 and IL-34 in TNBC. We thought that by investigating the relationship between IL-34 and ERBB2, we could identify TNBC groups for which immune checkpoint inhibitors are more effective. Using TNBC CEL files in the GSE database, the relationship among the gene expression of programmed death-ligand1 (PD-L1), IL-34, and ERBB2 was investigated.

Publication and presentation information

This article was published as a preprint on SSRN on June 28, 2024. It was made available for immediate access but did not undergo peer review. The article has since been withdrawn. Additionally, the content of this article was presented as a poster at the 2024 Annual Meeting of the Japan Breast Cancer Society.

## Materials and methods

Patients and samples

This retrospective study was conducted on patients with early-stage (stage I-III) TNBC who underwent NAC followed by surgery at Osaka Rosai Hospital between January 2012 and December 2020. A total of 39 patients were selected for inclusion. Patients were excluded if NAC was discontinued due to adverse events or if they had insufficient clinical or pathological data. All patients had pre-treatment TNBC confirmed by ultrasonography-guided core biopsies and were staged according to the AJCC Cancer Staging Manual, 8th Edition. The medical records of these patients were reviewed to obtain clinicopathological data, including age, menopausal status, clinical tumor size (cT), clinical nodal status (cN), histological grade (HG), Ki67index, tumor-infiltrating lymphocytes (TILs), PD-L1 status, absolute lymphocyte counts (ALCs) and neutrophil-to-lymphocyte ratio (NLR) before NAC.

Tumor sample processing

Biopsy samples were obtained before the administration of NAC, and all tumor tissues were fixed in 10% buffered formaldehyde, embedded in paraffin, and sectioned for histopathological evaluation. Post-surgical specimens were also obtained after NAC and were similarly fixed, embedded, and sectioned. Residual tumors were evaluated histologically to assess the pathological response to NAC, including the rate of pCR.

Immunohistochemical analysis

We evaluated the expression of estrogen receptor, progesterone receptor, Ki67 index, and PD-L1 [20]. In particular, the PD-L1 score was determined following the manufacturer’s instructions [21], and our previous study suggested that patients with PD-L1 with the immune cell (IC) score and tumor cell (TC) score of ≥2 have different prognosis [20]. In this study, scores of ≥2 for ICs and TCs were considered positive. TILs were examined according to the recommendation of the International TILs Working Group [22]. Regarding HER2, an immunohistochemical (IHC) assay was performed using the iVIEW™ DAB Detection Kit from Ventana (Tucson, AZ), and IHC scores were evaluated according to an algorithm adapted from the 2018 American Society of Clinical Oncology/College of American Pathologists (ASCO/CAP) HER2 testing in breast cancer guidelines [23].

Gene expression analysis

In total, 650 TNBC CEL files from the GSE database were extracted and analyzed [20]. Probe settings for ERBB2 and IL-34 were determined using Jetset as before 216836_s_at and 237046_x_at, respectively [24]. The expression value of ERBB2 above the median was determined to be ERBB2-high, and the expression value below the median was determined to be ERBB2-low.

Statistical analysis

R statistical software version 4.2.2 (http://www.r-project.org/) was used for statistical processing. Fisher’s exact test was used to compare the 2 × 2 and 2 × 3 groups. The paired t-test was used to compare HER2 expression levels before and after NAC. Cox hazards ratio and log-rank test were used to analyze prognosis. In the prognostic analysis using the Cox hazards ratio, multivariate analysis was performed for factors that showed significant univariate differences. Gene expressions in the two groups were compared using the Mann-Whitney U test. All statistical analyses were two-sided, and p-values <0.05 were considered significant.

## Results

Clinicopathological characteristics of patients recruited according to the HER2 status

The HER2 status and other clinicopathological factors in 39 TNBC samples before NAC were examined (Table 1). The patients were aged 33-86 (median = 57) years at diagnosis. Only ALCs demonstrated a statistically significant correlation with HER2 status (p = 0.047) (Table 1), whereas other clinicopathological factors did not.

**Table 1:** Clinicopathological characteristics according to the HER2 status before NAC. Statistical analysis: Fisher’s exact test. ALC: absolute lymphocyte counts; cN: clinical nodal status; cT: clinical tumor size; HER2: human epidermal growth factor receptor 2; HG: histological grade; IC: immune cells; NAC: neoadjuvant chemotherapy; NLR: neutrophil-to-lymphocyte ratio; pCR: pathological complete response; PD-L1: programmed cell death ligand 1; TC: tumor cells; TILs: tumor-infiltrating lymphocytes

		HER2 expression			
Characteristics		0	1+	2+	P-value
Number of patients		18	12	9	
Menopause	Pre	6	3	5	
	Post	12	9	4	0.353
cT	1, 2	14	11	9	
	3, 4	4	1	0	0.406
cN	Negative	9	8	7	
	Positive	9	4	2	0.337
HG	1, 2	7	5	6	
	3	11	7	3	0.344
Ki67 index	<20	4	3	2	
	≥20	14	9	7	1.000
TILs	≦10	14	11	5	
	>10	4	1	4	0.205
PD-L1	IC < 2	15	9	7	
	IC ≧ 2	3	3	2	0.882
	TC < 2	14	6	7	
	TC ≧ 2	4	6	2	0.267
Effects of NAC	Non-pCR	11	11	5	
	pCR	7	1	4	0.110
ALC	<1,500	11	5	1	
	≥1,500	7	7	8	0.047
NLR	<3	11	7	5	
	≥3	7	5	4	1.000

When dividing HER2 status into HER2 (0) and HER2-low, no correlation was observed between clinicopathological factors (Table 2).

**Table 2:** Clinicopathological characteristics according to the HER2 status before NAC (HER (0) vs. HER2-low). Statistical analysis: Fisher’s exact test. ALC: absolute lymphocyte counts; cN: clinical nodal status; cT: clinical tumor size; HER2: human epidermal growth factor receptor 2; HG: histological grade; IC: immune cells; NAC: neoadjuvant chemotherapy; NLR: neutrophil-to-lymphocyte ratio; pCR: pathological complete response; PD-L1: programmed cell death ligand 1; TC: tumor cells; TILs: tumor-infiltrating lymphocytes

		HER2 status		
Characteristics		0	low	P-value
Number of patients		18	21	
Menopause	Pre	6	8	
	Post	12	13	1.000
cT	1, 2	14	20	
	3, 4	4	1	0.162
cN	Negative	9	15	
	Positive	9	6	0.203
HG	1, 2	7	11	
	3	11	10	0.523
Ki67 index	<20	4	5	
	≥20	14	16	1.000
TILs	≦10	14	16	
	>10	4	5	1.000
PD-L1	IC < 2	15	16	
	IC ≧ 2	3	5	0.702
	TC < 2	14	13	
	TC ≧ 2	4	8	0.322
Effects of NAC	Non-pCR	11	16	
	pCR	7	5	0.488
ALC	<1,500	11	6	
	≥1,500	7	15	0.057
NLR	<3	11	12	
	≥3	7	9	1.000

Relationship of HER2 status with the pCR rate and prognosis

**Figure 1:**
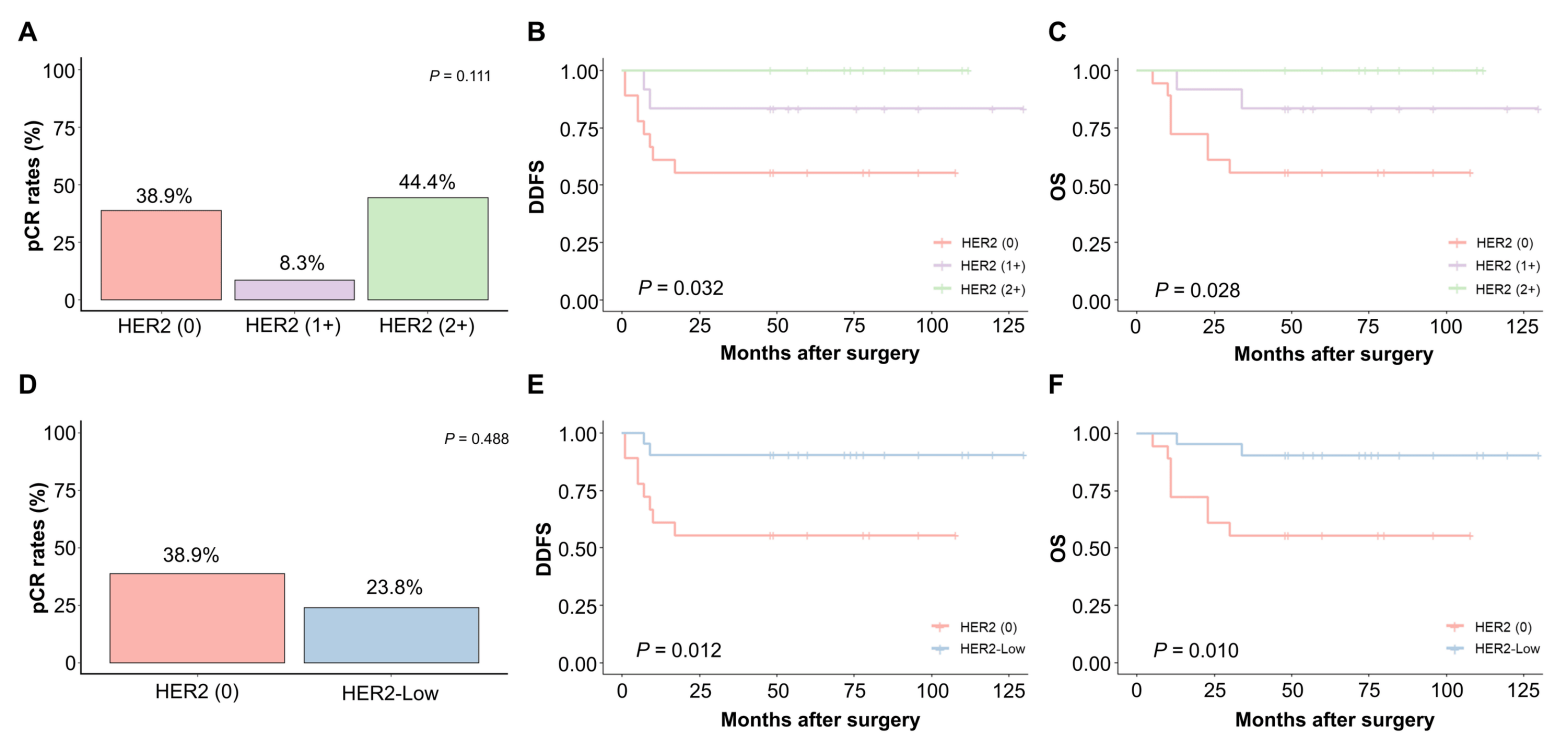
pCR rates and prognosis according to the HER2 status. (A) pCR rates for NAC according to the HER2 status. (B, C) DDFS and OS according to the HER2 status before NAC. (D) pCR rates for NAC according to the HER2 status: HER2 (0) vs. HER2-low. (E, F) DDFS and OS according to the HER2 status before NAC, HER2 (0) vs. HER2-low. Statistical analysis: Fisher’s exact test (A, D) and log-rank test (B, C, E, F). DDFS: distant disease-free survival; HER2: human epidermal growth factor receptor 2; NAC: neoadjuvant chemotherapy; OS: overall survival; pCR: pathological complete response

The pCR rates after NAC for HER2 states (0), (1+), and (2+) in IHC were 38.9% (7/18), 8.3% (1/12), and 44.4% (4/9), respectively, and no statistically significant difference was observed (p = 0.111) (Figure 1A). Conversely, samples with high HER2 status before NAC tended to have a poor prognosis based on distant disease-free survival (DDFS) and overall survival (OS) (p = 0.032 and p = 0.028, respectively) (Figures 1B, 1C). When HER2 (1+) and HER2 (2+) were defined as HER2-low, the pCR rate for HER2-low was 23.8% (5/21). Although no statistically significant difference was observed, the pCR rate for HER2-low was lower than that for HER2 (0) (Figure 1D). Moreover, HER2 (0) had a worse prognosis than HER2-low in both DDFS and OS (p = 0.012, p = 0.010, respectively) (Figures 1E, 1F). In the prognostic analysis using the Cox hazards ratio model, cT, cN, HER2, and PD-L1 (IC ≥ 2 and TC ≥2) showed statistically significant differences in both univariate and multivariate analyses (Table 3). In the univariate and multivariate analyses, HER2-low was a better prognostic factor for DDFS than HER2 (0) (p = 0.026; hazard ratio = 0.172; 95% confidence interval = 0.037-0.813; p = 0.049; hazard ratio = 0.197; 95% confidence interval = 0.039-0.992). Similar results were obtained for OS (p = 0.023; hazard ratio = 0.165; 95% confidence interval = 0.035-0.778; p = 0.034; hazard ratio = 0.161; 95% confidence interval = 0.030-0.869) (Table 3).

**Table 3:** Univariate and multivariate analyses of variables associated with DDFS and OS in TNBC. Statistical analysis: hazard ratio test. Multivariate analysis was performed using factors that showed significant differences in univariate analysis. ALC: absolute lymphocyte counts; cN: clinical nodal status; CI: confidence interval; cT: clinical tumor size; DDFS: distant disease-free survival; HG: histological grade; HR: hazard ratio; IC: immune cells; NAC: neoadjuvant chemotherapy; NLR: neutrophil-to-lymphocyte ratio; OS: overall survival; pCR: pathological complete response; PD-L1: programmed cell death ligand 1; TC: tumor cells; TILs: tumor-infiltrating lymphocytes

	Variable	Reference group	DDFS				OS			
			Univariate		Multivariate		Univariate		Multivariate	
Characteristics			HR (95% CI)	P-value	HR (95% CI)	P-value	HR (95% CI)	P-value	HR (95% CI)	P-value
Menopause	Pre	Post	1.24 (0.350–4.40)	0.737			1.239 (0.349–4.39)	0.740		
cT	3, 4	1, 2	10.6 (2.83–39.7)	4.63e-4	14.5 (1.85–113)	0.011	5.03 (0.581–43.5)	6.00e-4 (2.65–35.3)	2.44 (1.67–78.4)	0.013
cN	Positive	Negative	8.76 (1.85–41.5)	0.006	3.26 (0.947–34.5)	0.188	5.86 (1.06–32.3)	4.58e-3 (2.01–45.2)	5.72 (0.947–34.5)	0.057
HG	3	1, 2	1.34 (0.378–4.76)	0.649			1.25 (0.352-4.42)	0.734		
HER2	low	0	0.172 (0.037–0.813)	0.026	0.197 (0.039–0.992)	0.049	0.165 (0.035–0.778)	0.023	0.161 (0.030–0.869)	0.034
Ki67 index	≥20	<20	3.16 (0.400–24.9)	0.276			3.16 (0.400–24.9)	0.276		
TILs	>10	≦10	0.680 (0.144–3.21)	0.626			0.704 (0.149–3.32)	0.657		
PD-L1	IC; positive	IC; negative	1.25 (0.361–4.31)	0.728			1.23 (0.357–4.26)	0.742		
	TC; positive	TC; negative	1.24 (0.349–4.39)	0.741			1.20 (0.337–4.24)	0.783		
	IC ≥ 2 and TC ≥ 2	Others	3.82 (1.07–13.6)	0.039	8.77 (1.54–50.1)	0.015	3.78 (1.06–13.5)	0.040	14.6 (2.30–92.4)	0.004
ALC	≥1,500	<1,500	0.306 (0.079–1.19)	0.087			0.293 (0.076–1.14)	0.077		
NLR	≥3	<3	2.51 (0.708–8.91)	0.154			2.57 (0.725–9.14)	0.144		

Changes in the HER2 status before and after NAC

**Figure 2:**
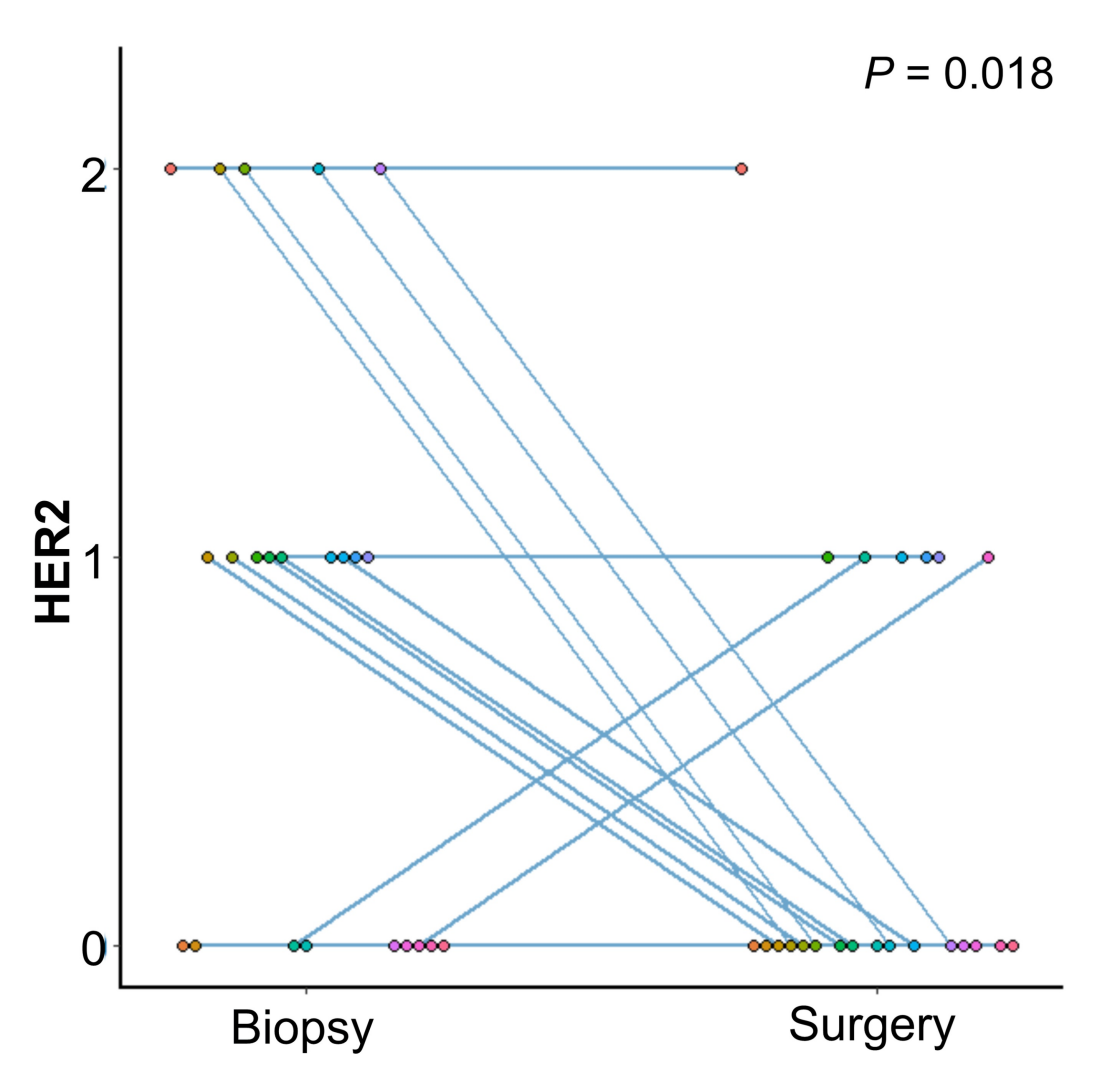
Alterations in the HER2 status before and after NAC. Statistical analysis: paired t-test. HER2: human epidermal growth factor receptor 2; NAC: neoadjuvant chemotherapy

A total of 23 samples had a residual tumor in surgical specimens after NAC. In these 23 samples, the HER2 status before and after NAC uniformly decreased (p = 0.018) (Figure 2).

Relationship between gene expressions in ERBB2-low and ERBB2-high

**Figure 3:**
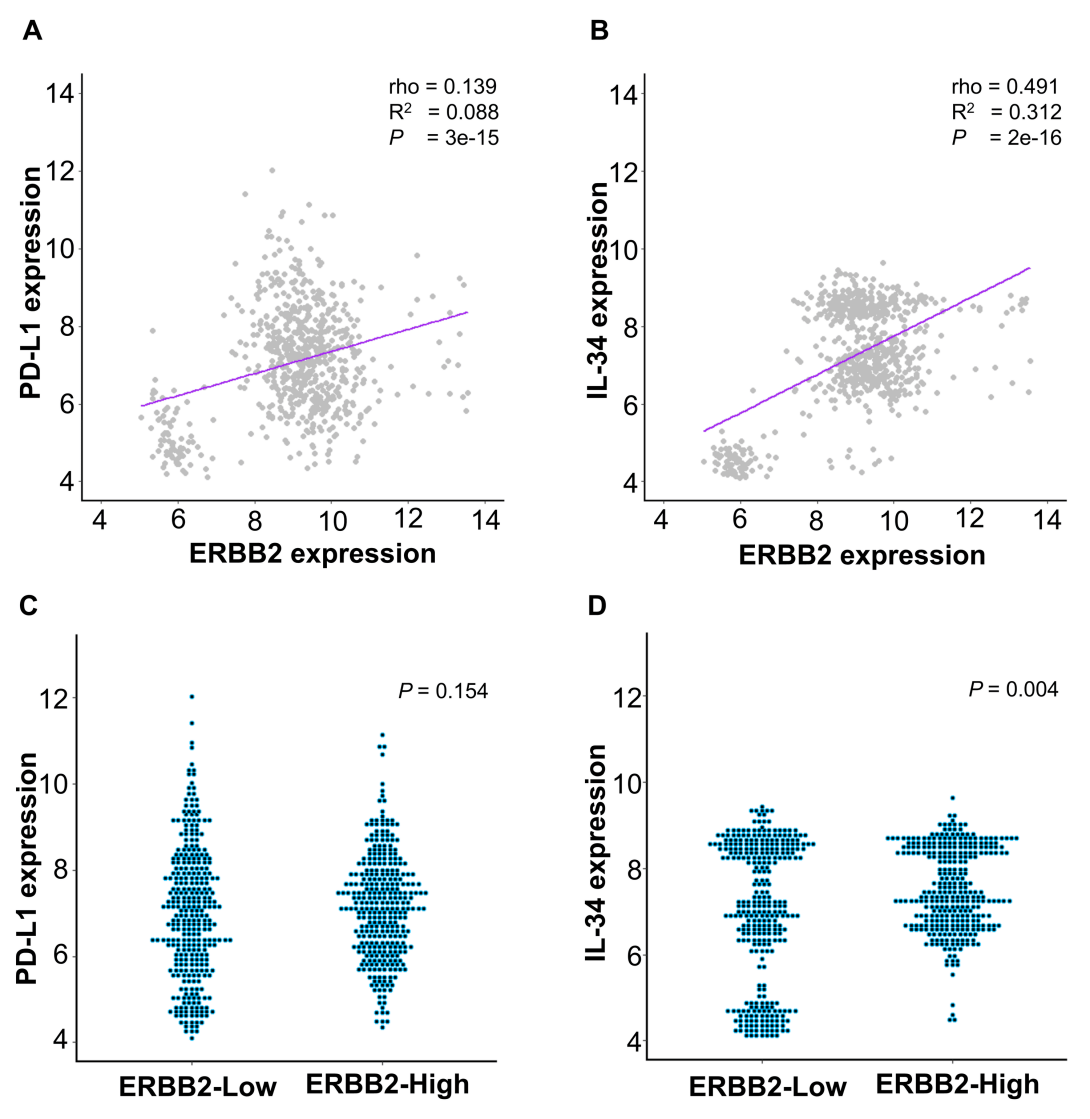
Relationship among gene expressions of PD-L1, IL-34, and ERBB2. (A) Scatter plots of PD-L1 gene expression that presented with ERBB2 expression. (B) Scatter plots of IL-34 gene expression that presented with ERBB2 expression. (C) Plots of PD-L1 gene expression in ERBB2-low and ERBB2-high. (D) Plots of IL-34 gene expressions in ERBB2-low and ERBB2-high. The values above and below the median of ERBB2 were designated as ERBB2-high and ERBB2-low, respectively. Statistical analysis: Spearman’s rank correlation coefficient (A, B) and Mann-WhitneyU test. IL-34: interleukin 34; PD-L1: programmed cell death ligand 1

No correlation was observed between PD-L1 and ERBB2 gene expression (rho = 0.139, R^2^ = 0.088, p = 3e-15) (Figure 3A); however, a weak positive correlation was observed between IL-34 and ERBB2 gene expression in 650 TNBC cases (rho = 0.491, R^2^ = 0.312, p = 2e-16) (Figure 3B). Furthermore, no significant difference in PD-L1 expression was found between the two groups ERBB2-low and ERBB2-high; however, the gene expression of IL-34 was higher in ERBB2-high, showing a statistically significant difference (p = 0.154 and p = 0.004, respectively) (Figure 3C, 3D).

## Discussion

In this study, we investigated the characteristics and prognosis of HER2 status in early-stage TNBC treated with A/T. Several studies have reported HER2 expression in TNBC. In this study, we analyzed HER2 expression, its clinicopathological factors, and prognosis in 39 patients with TNBC who underwent surgery after NAC at Osaka Rosai Hospital. No correlation was found among clinicopathological factors between HER2 (0) and HER2-low, and HER2 (0) had a worse prognosis than HER2-low. In addition, no correlation was found among clinicopathological factors, and no difference in the pCR rates was noted between HER2 (0) and HER2-low; however, HER2 (0) had a worse prognosis than HER2. Some studies have revealed that HER2 (0) [13,15] and HER2-low are associated with a better prognosis [16,17]. A recent meta-analysis was conducted [14]; however, a sufficient consensus has not been reached. The significance of HER2 expression may differ between early-stage TNBC and TNBC that has metastatically relapsed. Among previous studies, the study by Ma et al. analyzed a dataset that was most similar to the data analyzed in the present study, and the results were consistent, revealing that HER2-low was associated with a better prognosis than HER2 (0) [17]. High PD-L1 expression in early-stage TNBC was found to be associated with a poor prognosis [20], and this result agreed with the present finding after additional cases and observation periods. The present study further showed that HER2 (0) is also an independent factor for poor prognosis in DDFS and OS (p = 0.049, p = 0.034, respectively) (Table 3). Recently, we have been able to administer pembrolizumab for the treatment of early-stage TNBC. Improvements in pCR rates and event-free survivals have been reported after the addition of pembrolizumab to conventional chemotherapy [6]. However, immune checkpoint inhibitors such as pembrolizumab carry the risk of immune-related adverse events, which may be a factor in general physicians’ hesitation in administering them to their patients [25]. There may be more hesitation in treating patients with early-stage TNBC than patients with stage IV TNBC, who have an extremely poor prognosis and limited available treatment options. However, the direction changes when we know which groups of patients with early-stage TNBC have a poor prognosis. Based on the results of this study, TNBC-HER2 (0) maybe treated with chemotherapy supplemented with an immune check inhibitor rather than with traditional A/T chemotherapy. Kajiwara et al. reported that IL-34 is not only a poor prognostic factor in TNBC but also a factor that inhibits the effect of immune checkpoint inhibitors [18,19]. Therefore, to investigate the relationship between HER2 expression and PD-L1 and IL-34, we performed genetic analysis on 650 TNBC cases from the GSE dataset and determined the expression values of PD-L1 and IL-34 between the two groups divided by the median ERBB2. No significant difference in PD-L1 gene expression was found between the ERBB2-low and ERBB2-high groups; however, a difference in IL-34 gene expression was noted. The gene expression level of IL-34 was lower in the ERBB2-low group, showing a statistically significant difference (p = 0.004) (Figure 3D). For early-stage TNBC, pembrolizumab appears to be more effective in patients with PD-L1 expression than in those without PD-L1 expression [6]. Taking these into consideration, the efficacy of pembrolizumab against TNBC-HER2 (0) may not be low.

In this study, changes in HER2 expression before and after NAC due to A/T were also examined. HER2 expression tended to decrease uniformly (p = 0.018) (Figure 2). Several studies have reported on the reduction of HER2 expression during chemotherapy [26,27]. Although HER2 expression tended to decrease, these studies have focused on HER2-positive breast cancers and were combined with anti-HER2 therapy, which differs from the design of this study. Why NAC caused a decrease in the HER2 status in TNBC is unclear; however, the use of NAC-containing anthracyclines that target TOP2A, which is located near ERBB2, may explain this result. When administering T-DXd to relapsed TNBC, whether it is HER2-low must be confirmed. Considering the results of our study, if it is unclear whether HER2 should be measured in a pre-NAC biopsy sample or a post-NAC surgical sample, the pre-NAC biopsy sample may be more likely to be positive. Of course, HER2 staining results in biopsies before NAC may not be predictive of the effects of T-DXd. However, for relapsed TNBC, with limited treatment options, increasing treatment options may not be a bad thing. In addition, we used the iVIEW™ DAB Detection Kit from Ventana, which has been used at our hospital for some time, in the staining for HER2 status evaluation. HER2-low, as a companion test to T-DXd, requires the use of the ultraView Universal DAB Detection Kit from Ventana [12]. Although it has been reported for gastric cancer, the staining results of both kits are nearly the same [28].

Study limitations

This study was conducted retrospectively with a limited number of samples. In addition, the width of the 95% confidence interval in the results of the prognostic analysis was also large, presenting limitations.

## Conclusions

The results of this study suggested that the HER2 status in early-stage TNBC influences prognosis. Although the pCR rate with NAC for HER2 (0) was not low, the prognosis was poor. Chemotherapy with A/T alone may not be sufficiently effective for HER2 (0), and the addition of an immune checkpoint inhibitor may be more recommended. Furthermore, the HER2 status was reduced by the A/T chemotherapy. Thus, it may be helpful to consider this when testing for HER2-low expression. These observations should be confirmed in a future study with a larger number of TNBCs.

## References

[1] Siegel RL, Miller KD, Fuchs HE, Jemal A (2021). Cancer statistics, 2021. CA Cancer J Clin.

[2] Lin NU, Vanderplas A, Hughes ME (2012). Clinicopathologic features, patterns of recurrence, and survival among women with triple-negative breast cancer in the National Comprehensive Cancer Network. Cancer.

[3] Yang H, Wang R, Zeng F (2020). Impact of molecular subtypes on metastatic behavior and overall survival in patients with metastatic breast cancer: a single-center study combined with a large cohort study based on the Surveillance, Epidemiology and End Results database. Oncol Lett.

[4] Li X, Yang J, Peng L (2017). Triple-negative breast cancer has worse overall survival and cause-specific survival than non-triple-negative breast cancer. Breast Cancer Res Treat.

[5] Brown M, Tsodikov A, Bauer KR, Parise CA, Caggiano V (2008). The role of human epidermal growth factor receptor 2 in the survival of women with estrogen and progesterone receptor-negative, invasive breast cancer: the California Cancer Registry, 1999-2004. Cancer.

[6] Schmid P, Cortes J, Pusztai L (2020). Pembrolizumab for early triple-negative breast cancer. N Engl J Med.

[7] Gass P, Lux MP, Rauh C (2018). Prediction of pathological complete response and prognosis in patients with neoadjuvant treatment for triple-negative breast cancer. BMC Cancer.

[8] Sharma P, López-Tarruella S, García-Saenz JA (2018). Pathological response and survival in triple-negative breast cancer following neoadjuvant carboplatin plus docetaxel. Clin Cancer Res.

[9] Gabani P, Merfeld E, Srivastava AJ (2019). Predictors of locoregional recurrence after failure to achieve pathologic complete response to neoadjuvant chemotherapy in triple-negative breast cancer. J Natl Compr Canc Netw.

[10] Cortes J, Rugo HS, Cescon DW (2022). Pembrolizumab plus chemotherapy in advanced triple-negative breast cancer. N Engl J Med.

[11] Prat A, Bardia A, Curigliano G, Hammond ME, Loibl S, Tolaney SM, Viale G (2022). An overview of clinical development of agents for metastatic or advanced breast cancer without ERBB2 amplification (HER2-Low). JAMA Oncol.

[12] Modi S, Jacot W, Yamashita T (2022). Trastuzumab deruxtecan in previously treated HER2-low advanced breast cancer. N Engl J Med.

[13] Hu XE, Yang P, Chen S (2023). Clinical and biological heterogeneities in triple-negative breast cancer reveals a non-negligible role of HER2-low. Breast Cancer Res.

[14] Ergun Y, Akagunduz B, Karacin C, Turker S, Ucar G (2023). The effect of HER2-low status on pathological complete response and survival in triple-negative breast cancer: a systemic review and meta-analysis. Clin Breast Cancer.

[15] Li Y, Tsang JY, Tam F, Loong T, Tse GM (2023). Comprehensive characterization of HER2-low breast cancers: implications in prognosis and treatment. EBioMedicine.

[16] Atallah NM, Haque M, Quinn C (2023). Characterisation of luminal and triple-negative breast cancer with HER2 Low protein expression. Eur J Cancer.

[17] Ma Y, Jiao D, Zhang J, Lv M, Chen X, Liu Z (2024). HER2-low status was associated with better breast cancer-specific survival in early-stage triple-negative breast cancer. Oncologist.

[18] Kajihara N, Kitagawa F, Kobayashi T, Wada H, Otsuka R, Seino KI (2020). Interleukin-34 contributes to poor prognosis in triple-negative breast cancer. Breast Cancer.

[19] Kajihara N, Kobayashi T, Otsuka R (2023). Tumor-derived interleukin-34 creates an immunosuppressive and chemoresistant tumor microenvironment by modulating myeloid-derived suppressor cells in triple-negative breast cancer. Cancer Immunol Immunother.

[20] Imanishi S, Morishima H, Gotoh T (2022). Significance of the effects of chemotherapy on programmed death-ligand 1 expression in triple-negative breast cancer. Jpn J Clin Oncol.

[21] Vennapusa B, Baker B, Kowanetz M (2019). Development of a PD-L1 complementary diagnostic immunohistochemistry assay (SP142) for atezolizumab. Appl Immunohistochem Mol Morphol.

[22] Salgado R, Denkert C, Demaria S (2015). The evaluation of tumor-infiltrating lymphocytes (TILs) in breast cancer: recommendations by an International TILs Working Group 2014. Ann Oncol.

[23] Farshid G, Bilous M, Morey A (2019). ASCO/CAP 2018 breast cancer HER2 testing guidelines: summary of pertinent recommendations for practice in Australia. Pathology.

[24] Li Q, Birkbak NJ, Gyorffy B, Szallasi Z, Eklund AC (2011). Jetset: selecting the optimal microarray probe set to represent a gene. BMC Bioinformatics.

[25] Ye C (2023). Reconciling immunotherapy and autoimmunity: not for the faint of heart. Lancet Rheumatol.

[26] Ren X, Zhang X, Ma X (2023). Changes in HER2 status and survival outcomes in patients with non-pathological complete response after neoadjuvant targeted treatment. Medicine (Baltimore).

[27] Uczkowski D, Sekhri A, Gupta S, Gendler L, Misiukiewicz K, Levin M, Guerin B (2023). Treatment of HER2+ breast cancer: a retrospective of disease prognosis with loss of HER2 amplification on residual disease after neoadjuvant treatment in a community hospital setting. Am J Cancer Res.

[28] Yorozu K, Kurasawa M, Iino Y, Nakamura Y, Hashizume K, Harada N, Ochiai A (2023). Methods for preparing tissue microarray slides using xenografts with different levels of HER2 expression to standardize HER2 detection. Pathol Int.

